# Clinical significance of PET/CT uptake for peripheral clinical N0 non‐small cell lung cancer

**DOI:** 10.1002/cam4.2900

**Published:** 2020-02-13

**Authors:** Shuai Wang, Dong Lin, Xiaodong Yang, Cheng Zhan, Shihai Zhao, Rongkui Luo, Qun Wang, Lijie Tan

**Affiliations:** ^1^ Department of Thoracic Surgery Zhongshan Hospital Fudan University Shanghai China; ^2^ Department of Radiology Zhongshan Hospital Fudan University Shanghai China; ^3^ Department of Pathology Zhongshan Hospital Fudan University Shanghai China

**Keywords:** histology, NSCLC, PET, stage, survival

## Abstract

**Objective:**

In this cohort study, we determined the clinical value of the maximum standardized uptake value (SUVmax) of primary tumors in non‐small cell lung cancer (NSCLC).

**Study Design:**

A retrospective review of NSCLC patients was performed from January 2011 to December 2017. Peripheral cN0 NSCLC patients with tumor size ≤2 cm were included. SUVmax was calculated as a continuous variable for semiquantitative analyses. A receiver operating characteristic curve was analyzed to assess the cutoff threshold of SUVmax on pathological (p) nodal metastasis. We further evaluated the clinical relevance of SUVmax in peripheral cN0 NSCLC patients.

**Results:**

A total of 670 peripheral NSCLC patients with tumor size ≤2 cm were deemed cN0 by preoperative PET/CT scan. Statistical analyses suggested significant correlations of SUVmax with smoking status (*P* = .026), tumor volume (*P* = .001), pathology type (*P* = .008), tumor differentiation (*P* < .001), vessel invasion (*P* = .001), plural invasion (*P* < .001), pT stage (*P* < .001), nodal involvement (*P* < .001), and pathological tumor node metastasis stage (*P* < .001). A cutoff point of SUVmax of 3.8 (*P* < .001) could be used to predict pathological nodal metastasis. Multivariable analyses indicated that preoperative SUVmax >3.8 (odds ratio, 12.149; *P* < .001) was an independent predictor of nodal metastasis. Overall survival analyses further suggested that SUVmax was an independent prognostic indicator (hazard ratio, 2.050; *P* = .017).

**Conclusion:**

Preoperative SUVmax is a predictor of pathological nodal metastasis and prognosis for peripheral cN0 NSCLC patients with tumor size ≤2 cm. Our results indicate that assessment of PET SUVmax could improve stratification of these patients.

## Graphical abstract

Preoperative SUVmax of primary lesions is a predictor of pathological nodal metastases in peripheral cN0 NSCLC with tumor size ≤2 cm. Primary tumor SUVmax is associated with key clinicopathological features and prognosis of patients with peripheral cN0 NSCLC with tumor size ≤2 cm.

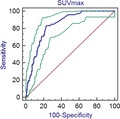

## 1 INTRODUCTION

In recent decades, multidisciplinary therapies for non‐small cell lung cancer (NSCLC) have greatly improved. However, prognosis of patients remains poor due to the aggressiveness of this disease.^1^ Peripheral clinical (c) N0 NSCLC with tumor size ≤2 cm is particularly interesting and standard management has not been well‐established. Based on our previous study, the clinical outcomes of these patients are heterogeneous, especially when definitive pathologic staging is lacking.^2^ Possible treatment modalities are varied, and include lobectomy, sublobar resection, and stereotactic body radiation therapy (SBRT), which demonstrates the ambivalence for peripheral cN0 NSCLC.^3^, ^4^ Peripheral cN0 NSCLC patients with tumor size ≤2 cm have a 70% 5‐year survival rate, suggesting that many cN0 patients have lymph node metastasis.^5^, ^6^ There is a clear need for accurate prediction of nodal disease, which will aid in the design of new therapeutic strategies.

NSCLC is characterized by dysregulation of glucose metabolism. Glucose transporter (Glut) protein expression and hexokinase activity are both upregulated.^7^ By administration of 18F‐fluorodeoxyglucose (^18^F‐FDG), the dysfunction of glucose metabolism can be measured quantitatively in vivo.^8^ However, the significance of the maximum standardized uptake value (SUVmax) on FDG‐positron emission tomography (PET) remains controversial.^9^, ^10^, ^11^, ^12^ As the evidence for the clinical value of FDG uptake in NSCLC patients remains limited, this cohort study was performed in peripheral cN0 NSCLC patients with tumor size ≤2 cm. We further conducted receiver operating characteristic curve (ROC) analysis to clarify the reliability of SUVmax on evaluation of nodal metastasis. The aim of this study was to determine the clinical value of SUVmax in peripheral cN0 NSCLC patients with tumor size ≤2 cm.

## 2 METHODS

### 2.1 Patients selection

Approval was obtained from the Research Ethics Committee of Zhongshan Hospital, Fudan University. This study protocol met the Health Insurance Portability and Accountability Act compliance standards. All participants provided written informed consent or informed consent by telephone. The informed consent included the following statements: (a) All patients provided their clinical data and follow‐up information in a scientific research process. (b) Information on the researchers' roles, research processes, and individual examination results were provided. (c) All patients were free to refuse or agree. (d) The final thesis and possible significant elements of the research would be published; however, no individual respondents would be identified or identifiable. (e) The individual information would not be available to others. From January 2011 to December 2017, we screened patients with peripheral NSCLC who received surgery. The criteria of inclusion were as follows: (a) Primary NSCLC was confirmed by pathological examination. (b) Lobectomy with lymph node dissection was performed to achieve complete resection. (c) According to National Comprehensive Cancer Network (NCCN) guidelines, tumors were defined as peripheral if they were located in the outer third of the lung parenchyma on axial computed tomography (CT) images.^13^ (d) Tumors were deemed to be clinical node negative by independent blind radiologists. (e) Post‐operative histopathological examination suggested tumor stage was pT1a‐b or visceral pleura invasion (pT2a) and residual foci did not exist in the surgical margin. (f) Patients with preoperative PET/CT scanning were included in this study. Patients did not receive chemotherapy or radiotherapy prior to PET/CT scanning. Patients who received neoadjuvant treatment were also excluded. We excluded patients with centrally located tumors, small cell lung cancer, or history of malignant disease. Patients with sublobectomy or pneumonectomy were also excluded.

### 2.2 Surgical resection and definition

Lobectomy with systemic mediastinal and hilar lymph node dissection was performed by thoracotomy or video‐assisted thoracoscopic surgery. Detailed operation information has been previously reported.^2^, ^14^, ^15^ Station 4, 7, and 9 lymph nodes were dissected for right‐sided tumors, and station 5, 6, 7, and 9 lymph nodes were dissected for left‐sided tumors. Stations 10, 11, and 12 were resected separately and assessed pathologically. Tumor volume (V) was calculated as follows: V = π/6 × width^2^ (cm^2^) × length (cm). Tumor staging was based on the 8th edition of the tumor node metastasis (TNM) classification for lung cancer.^16^, ^17^, ^18^ Surgical specimens were routinely fixed, embedded, and stained, then examined by light microscopy. The histologic sections were analyzed for evidence of vessel invasion by hematoxylin‐eosin staining. The histology subtypes of tumors were pathologically diagnosed according to 2015 World Health Organization (WHO) criteria.^19^ Image examinations were retrospectively reviewed by independent blind radiologists to confirm clinical nodal status. Clinical N0 disease was defined as hilar or mediastinal nodes with FDG uptake no greater than the normal background activity of the mediastinal blood pool (SUVmax <2.5) and short‐axis diameter less than 10 mm and long axis diameter less than 15 mm on the PET/CT scan (Figure S1). Lymph nodes with SUVmax ≥2.5 were considered suspicious for metastasis, irrespective of size. Lymph nodes showing higher attenuation than great vessels in thorax or benign calcification were not regarded as being malignant, even though those nodes had high FDG uptake.

### 2.3 Imaging protocol

^18^F‐FDG PET/CT imaging was done with a dedicated PET/CT scanner (Discovery PET and Lightspeed VCT 64, GE, Healthcare), based on Consensus Recommendations of National Cancer Institute.^20^ All patients fasted for 6 hours prior to scanning. Patient preparation included a serum glucose level of less than 140 mg/dL. PET/CT imaging was performed 60 minutes after administration of ^18^F‐FDG and the injected dose was 350 MBq. A CT scan was performed for attenuation correction and localization.

Irregular regions of interest (ROIs) were placed over lesions with ^18^F‐FDG accumulation on PET images. The ROIs were located by the corresponding CT images. For semiquantitative analysis of ^18^F‐FDG uptake, SUVmax of the primary tumor was measured. SUV was calculated as follows: tumor activity concentration/(injected dose/body weight).^20^, ^21^

### 2.4 Statistical analysis

Continuous variables were analyzed by one‐way analysis of variance, and the results are expressed as mean ± standard deviation. Dichotomous variables were analyzed by Fisher's exact test. The survival time was estimated from operation to death or the last follow‐up date. The Kaplan–Meier method was used to obtained survival curves. Prognostic factors were identified by univariate log‐rank test. ROC analysis was performed to determine the SUVmax cutoff value. Univariate and multivariable logistic regression analysis was used to investigate the association of pre‐operative clinical factors with lymph node metastasis. Preoperative variables entered in the univariate logistic regression analysis included gender, age, smoking status, laterality, tumor volume, and SUVmax of the primary tumor. Only variables with *P*‐value < .1 were entered into the multivariable logistic regression analysis. Multivariable logistic regression analysis was performed using forced entry. Survival curves were calculated using the Kaplan–Meier method. Univariate log‐rank test and Cox regression model analysis were performed to identify prognostic factors. Variables entered in the log‐rank test included gender, age, laterality, tumor volume, differentiation, vessel invasion, plural invasion, pT stage, nodal involvement, pathological tumor node metastasis (pTNM) stage, SUVmax, and adjuvant chemotherapy. To rule out confounding factors, only variables with *P*‐value < .05 were entered into the multivariable Cox regression model with forced entry. Two‐sided *P*‐values < .05 were considered statistically significant.

## 3 RESULTS

### 3.1 Patient characteristics

**Figure 1:**
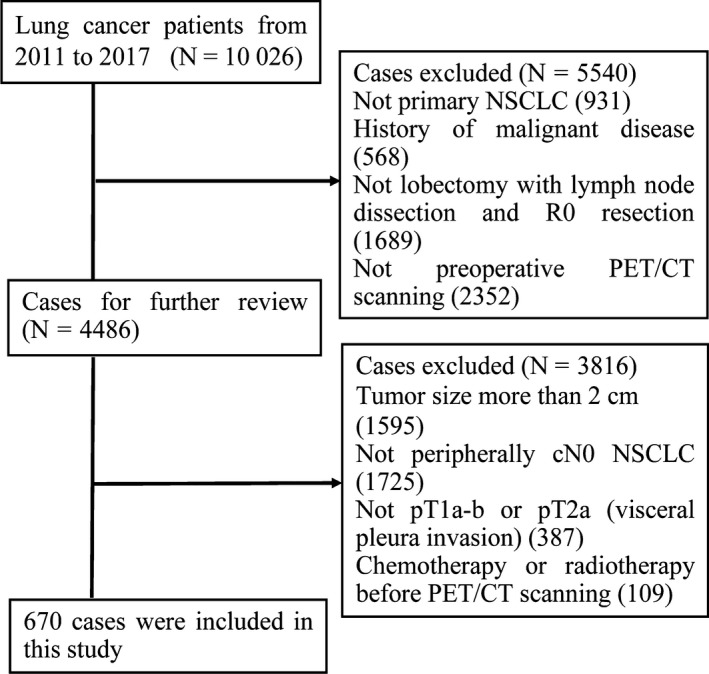
Flow diagram of patient selection

From January 2011 to December 2017, our lung cancer database included 10 026 cases, of which 9095 had pathological confirmation of primary NSCLC. According to the inclusion and exclusion criteria, 670 patients were included in this study. The case selection steps are summarized in Figure 1. Detailed clinical data of the 670 patients are shown in Table 1. The median age was 61 (range, 32‐84) years. The NSCLC cases were predominantly adenocarcinoma (n = 623, 92.9%). Of the 623 adenocarcinoma patients, the numbers of patients with minimally invasive adenocarcinoma (MIA), lepidic predominant (LEP), acinar predominant (ACI), papillary predominant (PAP), solid predominant (SOL), micropapillary predominant (MIP), and variants of invasive adenocarcinoma (VIA) were 42 (6.7%), 50 (8.0%), 317 (50.9%), 110 (17.7%), 48 (7.7%), 35 (5.6%), and 21 (3.4%), respectively.

**Table 1:** Clinicopathological features of 670 NSCLC patients

Characteristics	Number/percent (%)	SUVmax	*P*‐value
Age			
≥61 y	363 (54.2)	1.29 ± 0.46	.222
<61 y	307 (45.8)	1.34 ± 0.47	
Gender			
Male	302 (45.1)	1.39 ± 0.49	.109
Female	368 (54.9)	1.26 ± 0.44	
Smoking status			
Current/former	275 (41.0)	1.45 ± 0.42	.026
None	395 (59.0)	1.24 ± 0.41	
Tumor volume			
>2 cm^3^	264 (39.4)	1.48 ± 0.50	.001
≤2 cm^3^	406 (60.6)	1.20 ± 0.40	
Laterality			
Left	256 (38.2)	1.33 ± 0.47	.564
Right	414 (61.8)	1.31 ± 0.46	
Pathology			
Adenocarcinoma	623 (92.9)	1.30 ± 0.46	.008
Nonadenocarcinoma	47 (7.1)	1.49 ± 0.50	
Adenocarcinoma subtype			
MIA + LEP	92 (14.8)	1.61 ± 0.43	<.001
ACI + PAP	427 (68.5)	3.30 ± 0.51	
SOL + MIP + VIA	104 (16.7)	6.87 ± 0.94	
Differentiation			
G1	86 (12.8)	1.07 ± 0.25	<.001
G2	448 (66.9)	1.59 ± 0.45	
G3‐4	136 (20.3)	2.54 ± 0.51	
Vessel invasion			
Yes	48 (7.2)	1.76 ± 0.42	.001
No	622 (92.8)	1.30 ± 0.45	
Plural invasion			
Yes	185 (27.6)	1.86 ± 0.50	<.001
No	485 (72.4)	1.26 ± 0.43	
pT stage			
T1a	145 (21.6)	1.12 ± 0.32	<.001
T1b	240 (35.8)	1.42 ± 0.46	
T2a	185 (27.6)	1.86 ± 0.50	
Nodal involvement			
Yes	72 (10.7)	3.83 ± 0.67	<.001
No	598 (89.3)	1.25 ± 0.41	
TNM stage			
IA1	141 (21.1)	1.10 ± 0.20	<.001
IA2	309 (46.1)	1.62 ± 0.41	
IB	148 (22.1)	2.57 ± 0.45	
IIB + IIIA	72 (10.7)	3.83 ± 0.67	

Note

Data of SUVmax are expressed as mean ± standard deviation.

Abbreviations: ACI, acinar predominant; LEP, lepidic predominant; MIA, minimally invasive adenocarcinoma; MIP, micropapillary predominant; PAP, papillary predominant; SOL, solid predominant; TNM, tumor node metastasis; VIA, variants of invasive adenocarcinoma.

For clinical N0 patients, pathologic upstaging due to nodal metastasis occurred in 72 (10.7%) cases. Pathologic‐node positive patients were pN1a in 20 cases (3.0%), pN1b in seven cases (1.0%), pN2a1 in 14 cases (2.1%), pN2a2 in nine cases (1.3%), and pN2b in 22 cases (3.3%). According to the 8th edition of the TNM staging system, 141 patients had stage IA1 disease (21.0%), 309 had stage IA2 (46.1%), 148 had stage IB (22.1%), 27 had stage IIB (4.1%), and 45 had stage IIIA (6.7%). There were 79 patients (58 IIB or IIIA stage patients and 21 IB stage patients) who received adjuvant chemotherapy, while 591 patients did not receive adjuvant chemotherapy.

### 3.2 Clinical significance of SUVmax

As shown in Table 1, the SUVmax of primary lesions was significantly correlated with smoking status (*P* = .026), tumor volume (*P* = .001), pathology types of NSCLC (*P* = .008), tumor differentiation (*P* < .001), vessel invasion (*P* = .001), plural invasion (*P* < .001), pT stage (*P* < .001), nodal involvement (*P* < .001), and pTNM stage (*P* < .001), but not with age (*P* = .222), gender (*P* = .109), or laterality (*P* = .564).

All lung adenocarcinoma patients were broadly grouped into low‐, middle‐, or high‐grade histopathological subgroups based on aggressiveness.^19^, ^22^ The low‐grade histopathological subgroup included MIA and LEP. The middle‐grade histopathological subgroup included ACI and PAP, and the high‐grade histopathological subgroup included SOL, MIP, and VIA. The SUVmax values of the primary tumors were 1.61 ± 0.43, 3.30 ± 0.51, and 6.87 ± 0.94 for the low‐, middle‐, and high‐grade histopathological subgroups. The difference of SUVmax among histopathological subgroups was statistically significant (*F* = 73.561, *P* < .001) in lung adenocarcinomas (Table 1).

### 3.3 Prediction of nodal metastasis

**Figure 2:**
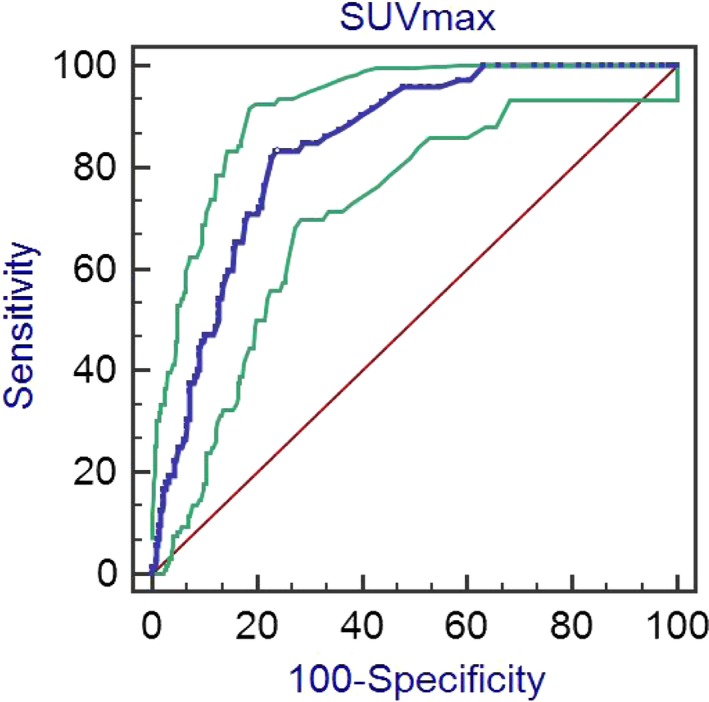
Receiver operating characteristic curve for prediction of pathological nodal involvement by the maximum standardized uptake value (SUVmax). The cutoff value with the best combined sensitivity and specificity was 3.8, with an area under the curve of 0.842. Blue lines indicate sensitivity and specificity of SUVmax for nodal metastasis prediction. Green lines indicate 95% confidence interval of sensitivity and specificity. Red line is the reference line

Of 670 clinical N0 patients, 72 patients had pathological nodal metastasis. The SUVmax of primary tumors in nodal metastasis patients was significantly higher than that of pN0 patients (3.83 vs 1.25, *P* < .001). According to the ROC curve (Figure 2), the threshold value of 3.8 was the closest to the point with both maximum sensitivity (83.33%; 95% confidence interval [CI], 72.7%‐91.1%) and specificity (77.26%; 95% CI, 73.7%‐80.6%), and was thereby selected as the cutoff value. The area under the curve (AUC) was 0.842 (95% CI, 0.812‐0.868, *P* < .001) with a Z statistic of 17.060. In this cohort, nodal metastasis occurred in 28.4% (60 of 211) of patients with SUVmax >3.8 compared with only 2.6% (12 of 459) of cases with SUVmax ≤3.8. Moreover, the incidence of pN2 (65.0%, 39/60) in patients with SUVmax >3.8 was higher than those with SUVmax ≤3.8 (50.0%, 6/12).

Univariate analysis revealed four preoperative factors that may be associated with nodal metastasis: sex, smoking status, tumor volume, and SUVmax (Table 2). Multivariable analysis was performed to gauge the potential roles of four preoperative factors on nodal upstaging. PET SUVmax of the primary tumor >3.8 (odds ratio [OR] 12.149; 95% CI, 6.234‐23.677, *P* < .001) was identified as the only independent preoperative predictor of nodal metastasis by multivariate analysis.

**Table 2:** Pre‐operative clinical features predict pathological nodal metastases

Variables	Univariate predictors		Multivariate predictors	
	OR (95% CI)	*P*	OR (95% CI)	*P*
Age (≥61 y vs <61 y)	1.288 (0.783‐2.119)	.319	—	—
Sex (male vs female)	0.624 (0.381‐1.020)	.060	0.903 (0.525‐1.554)	.713
Smoking status (current/former vs none)	1.645 (0.951‐2.845)	.075	1.497 (0.853‐2.501)	.119
Laterality (left vs right)	1.515 (0.927‐2.477)	.097	1.600 (0.929‐2.755)	.090
Tumor volume (>2 cm^3^ vs ≤2 cm^3^)	1.904 (1.084‐3.345)	.025	1.736 (0.995‐3.030)	.052
SUVmax (>3.8 vs ≤3.8)	2.719 (1.218‐4.352)	<.001	12.149 (6.234‐23.677)	<.001

Note

Statistical analysis was performed using Logistic regression. Variables with *P* < .100 in the univariate analyses were examined in the multivariate analyses.

Abbreviations: CI, confidence interval; OR, odds ratio.

### 3.4 Survival analyses

**Figure 3:**
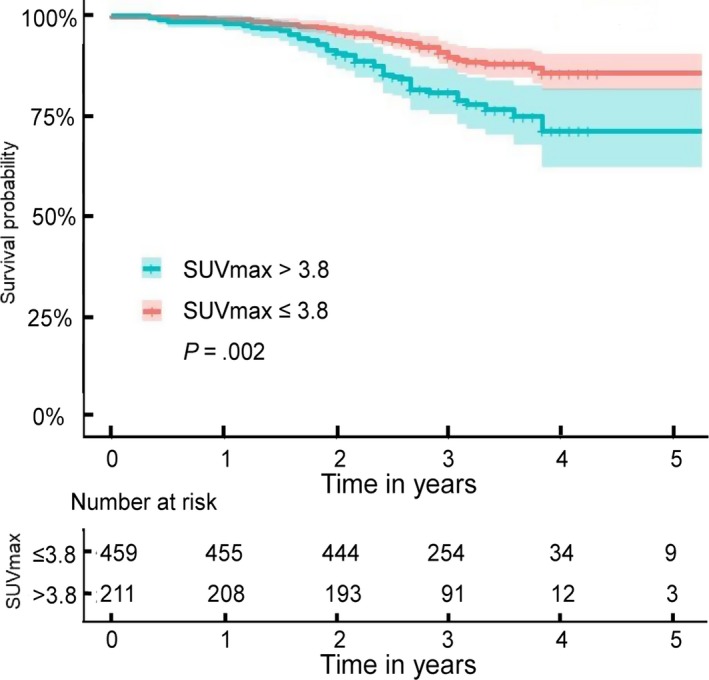
Overall survival according to SUVmax of primary tumors

The 1, 3, and 5‐year overall survival (OS) rates of the 670 patients were 98.8, 86.9, and 81.3%, respectively. The median survival time was 65.8 months. A value of 3.8 was selected as the cutoff value of the subgroup for these patients based on the ROC curve. For patients with SUVmax ≤3.8 (n = 459, 68.5%), the 1‐, 3‐, and 5‐year OS rates were 98.9, 88.9, and 85.7%, with a median survival time of 67.8 (95% CI, 66.0‐69.6) months. However, for patients with SUVmax >3.8 (n = 211, 31.5%), the 1‐, 3‐, and 5‐year OS rates were 97.6, 78.9, and 71.2%, with a median survival time of 60.5 (95% CI, 56.9‐64.1) months (Figure 3).

The log‐rank test suggested tumor volume (*P* = .011), differentiation (*P* = .046), vessel invasion (*P* = .018), plural invasion (*P* = .013), pT stage (*P* = .008), nodal involvement (*P* < .001), pTNM stage (*P* < .001), SUVmax (*P* = .002), and adjuvant chemotherapy (*P* = .022) as potential prognostic factors. However, gender (*P* = .055), age (*P* = .276), laterality (*P* = .626), and pathology (*P* = .926) did not reach statistical significance. To rule out confounding factors, we performed Cox proportional hazards model analysis. Multivariate analysis revealed that tumor volume (*P* = .029), plural invasion (*P* = .045), pT stage (*P* = .026), nodal involvement (*P* = .009), pTNM stage (*P* < .001), and SUVmax (*P* = .017) were independent prognostic factors (Table 3).

**Table 3:** Overall survival analyses of 670 NSCLC patients

Variable	Univariate analysis		Multivariate analysis		
	Chi‐Square	*P*	HR	95% CI	*P*
Age					
<61 y vs ≥61 y	1.189	.276	—	—	—
Gender					
Male vs Female	3.682	.055	—	—	—
Laterality					
Left vs right	0.237	.626	—	—	—
Pathology					
Ade vs NonAde	0.009	.926	—	—	—
Tumor volume					
>2 cm^3^ vs ≤2 cm^3^	8.238	.011	1.858	1.100‐2.551	.029
Differentiation					
G3 + G4 vs G1 + G2	5.873	.046	1.045	0.763‐1.913	.124
Vessel invasion					
Yes vs no	8.017	.018	1.604	0.982‐2.446	.052
Plural invasion					
Yes vs no	8.135	.013	1.619	1.011‐2.590	.045
pT stage					
T1b + T2a vs T1a	8.259	.008	1.947	1.107‐2.762	.026
Nodal involvement					
Yes vs no	13.369	<.001	2.112	1.173‐3.073	.009
pTNM stage					
IB + IIB + IIIA vs IA1 + IA2	16.616	<.001	2.268	1.260‐3.538	<.001
SUVmax					
>3.8 vs ≤3.8	9.099	.002	2.050	1.164‐2.976	.017
Chemotherapy					
Yes vs no	7.746	.022	1.556	0.881‐1.974	.063

Abbreviation: pTNM, pathological tumor node metastasis.

## 4 DISCUSSION

Management of NSCLC is stage‐dependent. The integrated PET/CT scan is the standard of NSCLC clinical staging according to NCCN guidelines.^13^ The specific goal of this study was to evaluate the clinical significance of the SUVmax of primary lesions in peripheral cN0 NSCLC patients with tumor size ≤2 cm.

We found correlations between the preoperative SUVmax of primary lesions and several significant clinicopathological features, including smoking status, tumor volume, pathology type, tumor differentiation, vessel invasion, pT stage, nodal involvement, and pTNM stage (Table 1). Our data are consistent with previous reports, which demonstrated that SUVmax was related to important clinicopathological characteristics in NSCLC.^8^, ^9^, ^10^, ^11^, ^12^ A better understanding of the molecular biology could explain the phenomena. NSCLC cells exhibit altered metabolism and consume excess glucose as an energy source. ^18^F‐FDG uptake is modulated by histologic factors in lung cancer. In this study, SUVmax was significantly higher in nonadenocarcinomas than in adenocarcinomas (1.49 vs 1.30, *P* = .008), which is consistent with a previous report.^23^ Overall, neuroendocrine lung tumors have been shown to exhibit a wide range of ^18^F‐FDG accumulation.^24^ It has also been shown that bronchioloalveolar carcinoma has relatively lower ^18^F‐FDG uptake.^25^ These phenomena can be explained by the varying expression levels of Glut and P‐glycoprotein.^23^, ^24^, ^25^ WHO updated the classification criteria of NSCLC in 2015.^19^ One interesting question that attracted our attention was whether ^18^F‐FDG uptake is heterogeneous in different histopathological subtypes of lung adenocarcinoma. In this cohort of 623 lung adenocarcinoma patients, we found that primary tumor SUVmax values in SOL, MIP, and VIA were the highest. SUVmax values in ACI and PAP were relatively low, while SUVmax values in MIA and LEP were the lowest (*F* = 73.561, *P* < .001). Previous studies have shown that histopathological subtypes are independent predictors of nodal metastasis, recurrence, and survival of lung adenocarcinoma patients.^19^, ^22^, ^26^ We demonstrated a close correlation of pretreatment SUVmax with histopathological subtypes in lung adenocarcinoma. Although the exact underlying mechanism remains unclear, these phenotypic observations suggest that ^18^F‐FDG accumulation has unrecognized roles in NSCLC.

Previous studies evaluating the prognostic significance of SUVmax in NSCLC have drawn different conclusions. Downey et al^11^ found that SUVmax was not an independent predictor of survival in patients with NSCLC. Hoang et al^27^ reported that SUVmax did not have a significant relationship with the survival of patients with advanced NSCLC. Vesselle et al^12^ found that SUVmax did not provide additional prognostic information in resectable NSCLC. However, most studies have reported that preoperative SUV is a significant prognostic factor in early stage (I and II) NSCLC.^6^, ^8^, ^9^, ^10^, ^23^, ^26^ The difference among these studies may be a result of patient selection, tumor stage, and a limited number of cases. Differences in the acquisition and interpretation of SUVmax may also lead to inconsistent results. Thus, it will be necessary to standardize PET/CT imaging modality, including patient preparation, image acquisition, image reconstruction, and quantitative image analysis. In this study, PET/CT imaging was performed based on the Consensus Recommendations of the National Cancer Institute.^20^ Our data indicated that NSCLC patients with high SUVmax had worse survival than those with low SUVmax. Our findings also indicated that assessment of primary tumor SUVmax might provide valuable information in terms of clinical outcomes and follow‐up management.

Mountainous studies have demonstrated the relatively high accuracy of PET/CT scanning for TNM staging in NSCLC.^13^, ^28^ However, based on our previous study,^2^ a subset of peripheral cN0 NSCLC patients with tumor size ≤2 cm will be upstaged due to pathologic nodal disease (Figure S2). In 670 cN0 patients, pathologic nodal upstaging occurred in 72 (10.7%) cases, with 20 cases of pN1a, seven cases of pN2a1, 14 cases of pN2a1, nine cases of pN2a2, and 22 cases of pN2b. Patients with pathologic nodal involvement had significantly higher SUVmax than those without pathologic nodal disease (*P* < .001; Table 1). It may be difficult to predict the status of nodal metastasis by preoperative SUVmax. We set the cutoff score of SUVmax based on ROC analyses, with maximum sensitivity (83.33%) and specificity (76.25%). Thereby, 3.8 was selected as the cutoff value, with an AUC of 0.842 (*P* < .001; Figure 2). This is particularly important as patients with SUVmax >3.8 had high risk of pathological nodal disease compared with patients with SUVmax ≤3.8 (28.4% vs 2.6%). Furthermore, pN2 was more frequent in patients with SUVmax >3.8 than those with SUVmax ≤3.8 (65.0% vs 50.0%).

Multivariate analysis also suggested that PET SUVmax >3.8 (*P* < .001) was the only independent preoperative predictor of nodal metastasis. Patients with high risk of nodal disease are candidates for lobectomy, which might achieve anatomic surgical resection with complete nodal dissection. Sublobectomy, including wedge resection and segmentectomy, is oncologically compromised resection of peribronchial, hilar, and mediastinal lymph nodes.^29^ Sublobectomy with low rates of nodal sampling could underestimate the tumor stage.^30^ Similarly, Robson et al^31^ reported that SBRT might be associated with significant regional control failure and worse outcomes as a result of occult pathological nodal metastasis. Therefore, it might be recommended that sublobectomy or SBRT be avoided for patients with SUVmax >3.8. Further diagnostic procedures, such as endobronchial ultrasound, might be required for those patients if sublobectomy or SBRT is being considered.

Previous studies have demonstrated an association of SUVmax with occult nodal disease in lung cancer.^32^, ^33^, ^34^, ^35^ Our findings add the following to the literature: (a) further validation of associations of SUVmax with N descriptors and stage groupings in the 8th edition of TNM classification; (b) the significance and heterogeneity of primary tumor SUVmax in different histopathological subtypes of lung adenocarcinoma, such as LEP, ACI, PAP, SOL, and MIP; (c) discriminative cutoff point for nodal disease at SUVmax of 3.8 using ROC analyses; (d) the only independent pre‐operative predictive value of SUVmax for pathological nodal metastasis; (e) independent prognostic value of SUVmax for patient survival; and (f) clinical significance of SUVmax in high selected patients with large sample volume (670 cases with peripheral cN0 NSCLC with tumor size ≤2 cm).

Our study had certain limitations. First, this was a retrospective single‐center study with selected cases. It would be important to evaluate intraparenchymal and hilar N1 node involvement. We only included patients undergoing anatomic lobectomy with systemic mediastinal and hilar lymph node dissection. Thus, there might be unavoidable bias toward those patients with greater likelihood of more aggressive disease. The relatively low percentage of patients who underwent PET/CT scanning also created a selection bias. Thus, the sample error and selection bias cannot be ignored. Second, information regarding post‐operative local recurrence and remote metastasis was limited. We only performed OS analyses without disease‐free survival. Therefore, the significance of SUVmax on disease‐free survival was not determined in this study. Adenocarcinomas and squamous cell carcinomas are inherently different in glucose metabolic activity. The existed error resulted from pathological difference could not be ignored. According to previous studies, absence of ground glass opacity is associated with lymph node metastasis in NSCLC.^36^, ^37^ CT manifestation of tumors could be used to predict pathological lymph node disease. So, we would collect disease‐free survival information and CT images data to further clarify significance of SUVmax in future research. On the other hand, patients included in this study were actually cN0 and the study was underpowered to analyze the survival difference. Third, we did not include central NSCLC data, although SUVmax had prognostic value in peripheral NSCLC. We assumed that selected peripheral NSCLC patients with primary tumor SUVmax ≤3.8 could be candidates for wedge resection or SBRT, because the risk of nodal metastasis is low (2.6%). While more invasive modalities, such as pre‐operative endobronchial ultrasound‐transbronchial needle aspiration or mediastinoscopy and lobectomy with lymphadenectomy, should be considered for patients with high primary tumor SUVmax (>3.8) because the frequency of pathologic nodal disease is high (28.4%). However, our assumption is likely to be tenuous at best and requires larger scale external validation. Metrics concerning the accuracy of nodal involvement prediction cannot indicate whether SUVmax will be useful in clinical practice. For a continuous variable of SUVmax, using a cutoff value of 3.8 might oversimplify the relationship. Future larger multicenter prospective studies are necessary to confirm our findings and multicenter randomized controlled trials with comparator arms will be needed to further guide clinical management.

## 5 CONCLUSIONS

In summary, this study demonstrates that primary tumor SUVmax is associated with key clinicopathological features and prognosis of patients with peripheral cN0 NSCLC. Importantly, we found that the SUVmax of primary lesions was significantly correlated with histopathological subtype and lymph node metastasis. Continuing research will be necessary to further confirm the clinical use of primary tumor SUVmax in NSCLC.

## CONFLICT OF INTEREST

None declared.

## AUTHOR CONTRIBUTION

Conceptualization: Shuai Wang and Lijie Tan; Methodology and validation: Dong Lin, Xiaodong Yang, Shihai Zhao, Rongkui Luo, Zhan Cheng; Data analysis: Shuai Wang Dong Lin, Xiaodong Yang, Shihai Zhao, and Rongkui Luo; Writing: Shuai Wang; Supervision: Qun Wang and Lijie Tan.

## Data Availability

The data used to support the findings of this study are available from the authors upon request.
