# Supplementary figures and images for: Clinical significance of PET/CT uptake for peripheral clinical N0 non‐small cell lung cancer
Source: Cancer Med. 2020 Feb 13;9(7):2445–53. doi: 10.1002/cam4.2900 (PMC7131855; doi:10.1002/cam4.2900)

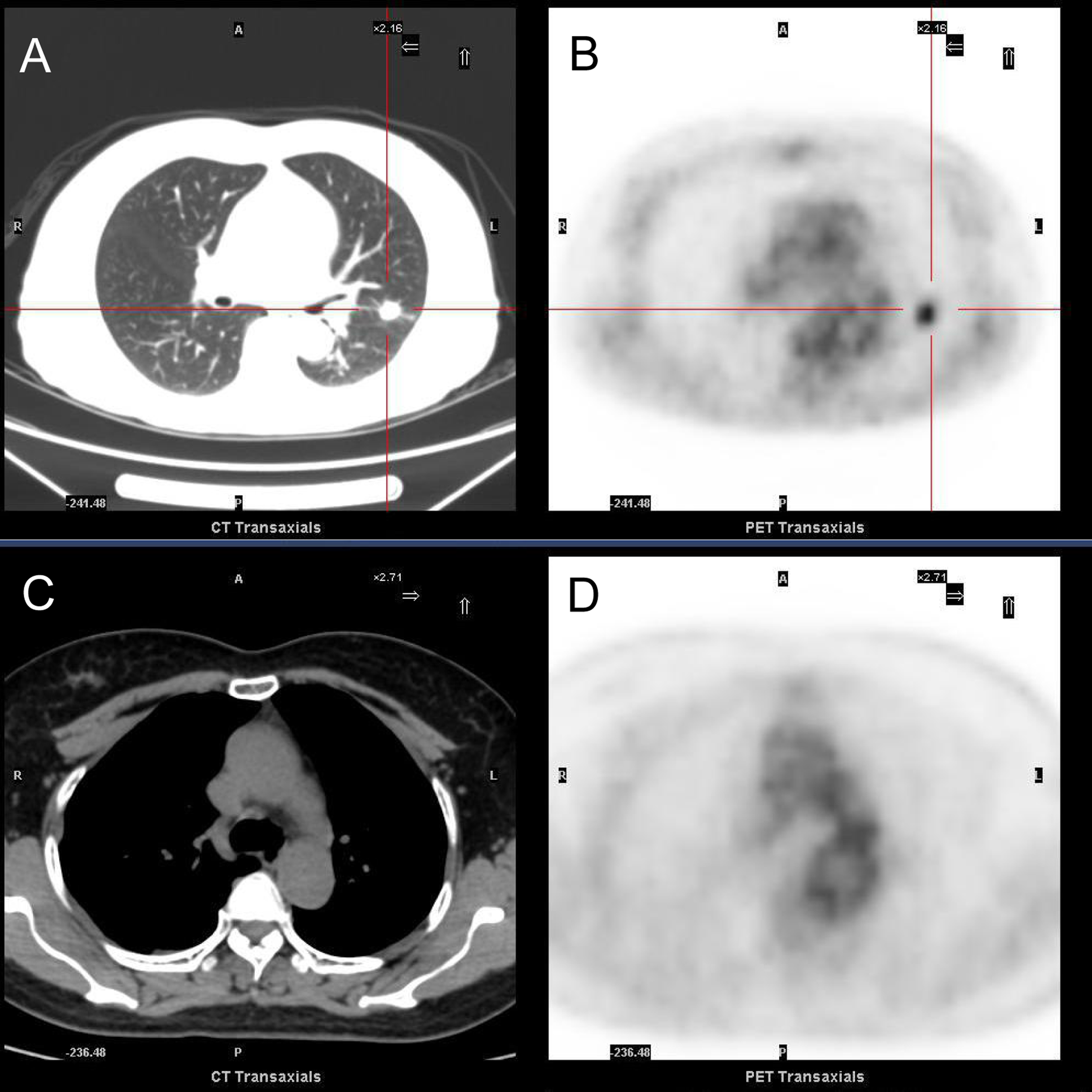

Supplement: Supplementary file 1 [file CAM4-9-2445-s001.tif]

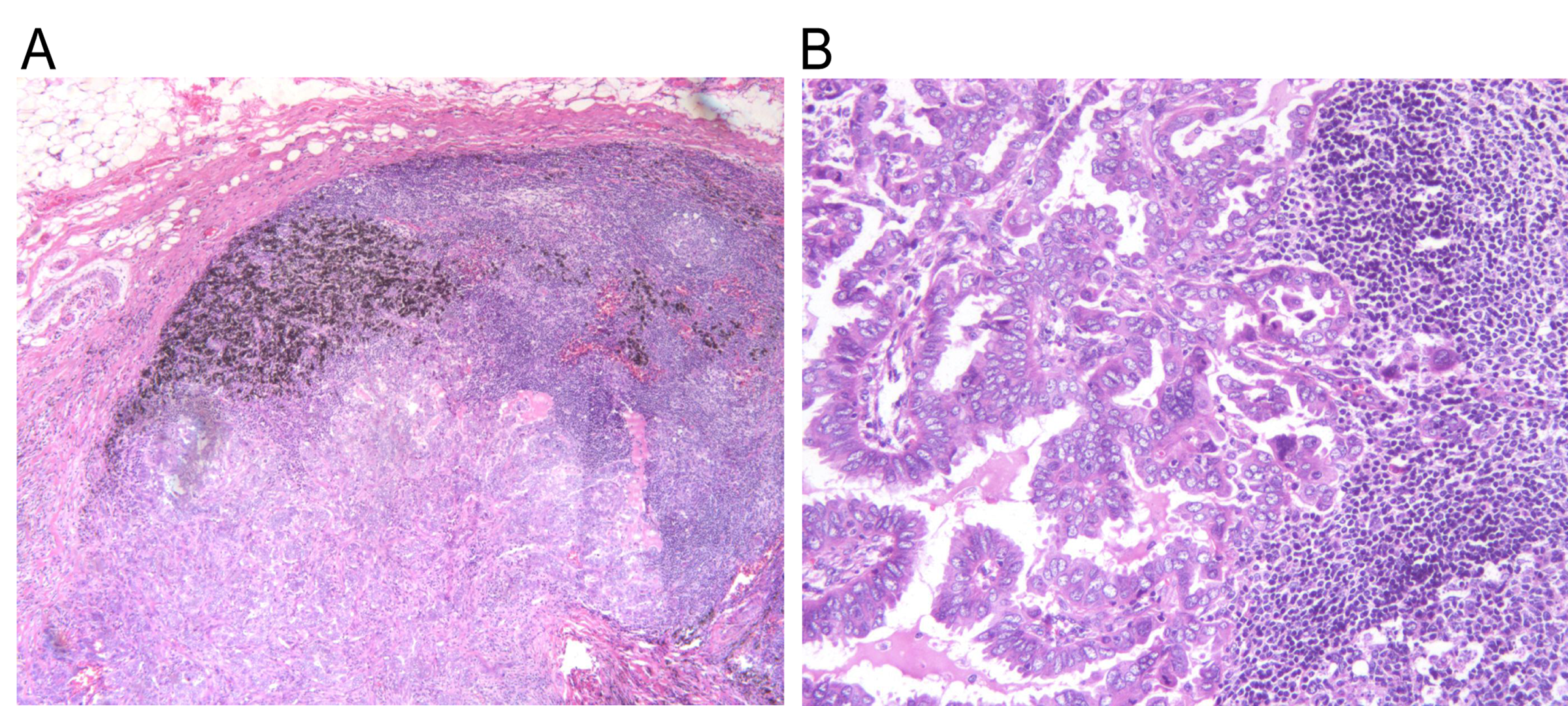

Supplement: Supplementary file 2 [file CAM4-9-2445-s002.tif]
